# Editorial: Advances in sport science: latest findings and new scientific proposals, volume II

**DOI:** 10.3389/fpsyg.2025.1550371

**Published:** 2025-01-21

**Authors:** Rubén Maneiro, Iyán Iván-Baragaño, José L. Losada, Antonio Ardá, Mario Amatria, Gudberg K. Jonsson

**Affiliations:** ^1^Faculty of Education and Sport, University of Vigo, Vigo, Spain; ^2^Department of Sport Sciences, Faculty of Medicine, Health and Sports, Universidad Europea de Madrid, Villaviciosa de Odón, Spain; ^3^Department of Social Psychology and Quantitative Phycology, University of Barcelona, Barcelona, Spain; ^4^Department of Physical and Sport Education, University of A Coruña, A Coruña, Spain; ^5^Department of Physical Education and Sport, Pontifical University of Salamanca, Salamanca, Spain; ^6^Social Science Research Institute & Human Behavior Laboratory, University of Iceland, Reykjavík, Iceland

**Keywords:** behavior analysis, performance analysis, sports psychology, innovative methodologies, women's sport, adapted sport

Research in Sport Science is shaped by technological advancements (Oviedo-Caro and Sánchez-Trigo, 2024), shifting trends, and social influences (Chan et al., 2019). In recent years, significant progress has been made in this field through various technologies, methodologies, and research approaches. Wearable sensors, increasingly accessible in both professional and amateur sports, have facilitated the collection of a vast amount of physiological data (Seshadri et al., 2019). In other sports, where financial investment is greater, technologies based on computer vision and neural networks have enabled athlete monitoring, producing data volumes capable of providing valuable insights into player performance (Komorowski and Kurzejamski, 2022). Simultaneously, there has been a rise in the prominence of women's sports in society, paralleled by an increase in academic publications aimed at analyzing various aspects related to women's sports (Martínez-Rosales et al., 2021). However, even today, areas such as performance analysis remain predominantly skewed toward men's sports (Kryger et al., 2022). In addition, in response to growing concerns and interest in mental health and associated factors, psychological variables in sports have become a recurring research theme (Reyes-Bossio et al., 2022). This highlights the need to approach sports in general, and athletic performance in particular, from a multidisciplinary perspective.

The multidisciplinary team, comprising professionals from dynamic sports communities and experts in research methodology, has not remained unaffected by the evolving changes and emerging trends within the field of Sport Science research. Consequently, the Research Topic *Advances in sport science: latest findings and new scientific proposals, volume II* has sought to address these new needs and trends in each of these areas. This Research Topic has aimed to strengthen Sport Science through diverse perspectives, such as physiology, technical-tactical performance, and sport psychology. Furthermore, it has created a favorable space for researchers to conduct studies in areas where scientific literature is limited, such as youth sports, women's sports, and adapted sports. The studies published within this Research Topic have expanded the body of knowledge, bringing researchers and practitioners closer to a more comprehensive understanding of their respective sports while narrowing the gap between theoretical knowledge and practical application.

## Contributions of the research articles

Sports practice is socially linked to gender, leading to social stigma and reduced participation. This presents a clear issue, considering the benefits associated with sports practice and physical exercise. Understanding the differences and similarities between men and women in sports participation is essential for advancing effective gender equality. Within this topic, research has been conducted on gender differences in managing competitive contexts among male and female archers. The findings revealed that women were more vulnerable to the negative effects of failure during competition. Moreover, there is a consensus on the positive impact of physical activity on attention levels, academic performance, and social relationships. However, very few studies have focused on analyzing this influence in students with special educational needs. In this Research Topic, a positive association between physical activity and these variables has been demonstrated in students with intellectual disabilities—a population requiring special attention and historically excluded from academic and research domains. This type of socially impactful work highlights the need for research in heterogeneous populations, encompassing the full spectrum of individuals, whether athletes or students. Finally, and in direct relation to sports performance, this Research Topic has highlighted the influence of psychological and personality-related variables on the professional performance of athletes across various sports. In football, differences in players' personalities have been demonstrated based on their level of experience and gender. This is undoubtedly linked to the individual motivations of each athlete, which, as evidenced, can regulate behavior and the degree of effort individuals exert. Similarly, the influence of social, psychological, and demographic variables on emotional regulation in individual athletes has been analyzed, yielding significant conclusions for the field of knowledge.

In summary, the second edition of “*Advances in sport science: latest findings and new scientific proposals*” serves as a valuable platform for researchers worldwide to exchange knowledge and disseminate novel research in the field of Sport Science. The findings presented, with significant social impact, have the potential to enhance practices and outcomes across diverse domains of application.

## Future research directions

To further advance the field of Sport Science, the third edition of “*Advances in sport science: latest findings and new scientific proposals” – volume III* will be launched. This volume will serve as a dedicated platform for the dissemination of diverse academic contributions, with a particular emphasis on:

- The influence of psychological factors on athletic skill development.
- The development and evaluation of innovative training methodologies.
- The application of advanced statistical techniques in sports research.

These areas of focus will drive continued progress and innovation within the field of Sport Science.
